# Editorial: Differences between emmetropic and myopic eyes: implications for myopia development, its progression, and ocular health

**DOI:** 10.3389/fmed.2025.1644062

**Published:** 2025-07-10

**Authors:** Pablo De Gracia, Fuensanta A. Vera-Diaz, Alexandra Benavente-Pérez, Pablo Pérez-Merino

**Affiliations:** ^1^University of Detroit Mercy School of Optometry, Novi, MI, United States; ^2^New England College of Optometry, Boston, MA, United States; ^3^SUNY College of Optometry, New York, NY, United States; ^4^Instituto de Óptica, Consejo Superior de Investigaciones Científicas, Madrid, Spain

**Keywords:** myopia development, axial elongation, environmental risk factors, scleral remodeling, retinal signaling pathways, peripheral blur anisotropy, retinal pigment epithelium (RPE), visual experience and myopia modulation

Myopia has emerged as a pressing public health concern worldwide of increasing prevalence that represents a growing burden due to the myopia-associated visual impairment. This, coupled with our expanding understanding of its complex etiology, underscores the urgent need for multidisciplinary research in myopia. This Research Topic of *Frontiers in Medicine* brings together nine original studies that traverse the full spectrum of myopia science—from large-scale epidemiological insights to innovative investigations of optical and molecular mechanisms. Together, they offer an integrated perspective on how we define, detect, and potentially intervene in the development and progression of myopia.

## Global and environmental perspectives on myopia

This Research Topic frames the global impact of myopia with three large-scale epidemiological studies. Mu et al. investigated changes in myopia prevalence among nearly 850,000 school-age children in Shenzhen, China, before, during, and after the COVID-19 pandemic. Their findings highlight the accelerating impact of behavioral shifts—specifically decreased outdoor activity and increased near work—on myopia. Complementing this, Li et al. presented a prospective study of nearly 38,000 children in Xuzhou, China, revealing semester-level myopia progression and emphasizing the vulnerability of early primary school students. Adding an environmental dimension, Liu T. et al. conducted a cross-sectional study linking satellite-derived measurements of outdoor artificial light at night (ALAN) to increased myopia prevalence in over 33,000 adolescents, identifying it as a modifiable environmental risk factor with implications for urban lighting policy.

## Structural and functional changes in the myopic eye

Three studies in this Research Topic explored structural and functional manifestations of myopia. Huang et al. compared macular characteristics in eyes with myopic choroidal neovascularization and their contralateral eyes, and propose that reduced perforating scleral vessels and choroidal thinning may be potential diagnostic markers for advanced myopia disease. Bueno et al. used second harmonic generation microscopy to study collagen remodeling in the posterior sclera of chicks with form-deprivation myopia, revealing increased collagen alignment without changes in thickness. Their use of objective Radon Transform-based quantification suggests that collagen architecture beyond overall scleral dimensions may play a key role in the biomechanical behavior of the myopic eye. Importantly, the study challenges prior assumptions that scleral thinning is a hallmark of axial elongation, instead emphasizing the importance of fiber orientation in ocular growth. These findings offer a new window into the role of the sclera in myopia and support the use of advanced imaging techniques to monitor tissue-level remodeling *in vivo*. Kendrick et al. introduced a Radial Asymmetry Metric to quantify peripheral blur anisotropy in myopia. Their study provides compelling evidence that blur asymmetry is not only more pronounced at greater retinal eccentricities but also differs in orientation in myopic eyes, with larger vertically oriented blur in the temporal retina. This framework represents an advance over traditional Modulation Transfer Function analyses by capturing both the direction and magnitude of blur that are otherwise missed. Their results point to the importance of peripheral image quality and its anisotropic properties in shaping ocular growth.

## Molecular and cellular mechanisms underpinning eye growth

Transitioning from macroscale to molecular insights, two studies in this topic aimed to elucidate the signaling pathways that govern myopia development. Palumaa et al. presented a meta-analysis of retinal transcriptome data from animal models, identifying both conserved and species-specific gene expression patterns—several of which overlap with human data. They unify data across multiple datasets from mice and chicks with experimental myopia, revealing consistent molecular responses not evident in individual studies. Among the conserved pathways, Transforming Growth Factor-beta signaling and circadian entrainment emerged as central regulators, while species-specific patterns highlighted dopamine signaling in mice and glucagon in chicks. The convergence between animal model data and human genetic findings adds translational weight to the molecular targets identified. The authors also underscored the value of meta-analytic strategies in enhancing reproducibility and discovering novel gene targets for future investigation. Sanchez et al. offered novel insights into the role of exosomes derived from the retinal pigment epithelium in tree shrews, revealing proteomic differences in myopic eyes through an innovative *ex vivo* model. Their profiling identified over 500 proteins, with dozens uniquely expressed in each group and 286 significantly differentially regulated. In myopic eyes, upregulated proteins were primarily associated with cytoskeletal remodeling, oxidative stress response, and extracellular matrix dynamics—aligned with active tissue remodeling. Conversely, non-myopic eyes showed enrichment of proteins involved in phototransduction and mitochondrial homeostasis. These findings support the hypothesis that RPE-derived exosomes modulate emmetropization through cell signaling and metabolic regulation, and suggest potential applications in biomarker discovery and future pharmacological interventions for myopia.

## Visual experience and experimental modulation

The last study of this Research Topic described how visual experience can be manipulated to influence ocular growth at a mechanistic level. Liu H. et al. investigated the effects of dynamic checkerboard visual stimuli—with different spatial and temporal frequencies—on ocular physiology and molecular signaling in chicks. Through a controlled experimental design with short-, medium-, and long-term exposure durations, the authors measured changes in choroidal thickness, ocular biometry, dopamine metabolite concentrations, and the expression of key genes linked to eye growth. Their findings reveal that exposure to 10 Hz flickering stimuli, particularly with ON-type patterns leads to greater elongation and myopia, and also correlates with increased levels of retinal dopamine metabolites. Additionally, gene expression analyses suggested an integrated visual, neurochemical, and genetic response. While choroidal thinning was an early feature in myopia, its predictive value for long-term growth was limited, indicating that multiple signaling pathways operate in concert. This study offers important evidence that the visual scene—its flicker rate, contrast polarity, and spatial granularity—can influence biological responses relevant to myopia, supporting the development of novel interventions to slow its progression.

## Conclusion

Taken together, the nine contributions to this Research Topic present a compelling, multifaceted picture of myopia. Large-scale epidemiological studies reveal alarming trends in prevalence and progression, along with environmental risk factors such as artificial light exposure. Structural and functional analyses underscore differences in scleral and choroidal architecture as well as peripheral blur asymmetry in myopic eyes. Molecular and proteomic investigations expose conserved signaling pathways across species and the role of exosomes in intraocular communication. Finally, experimental manipulation of visual stimuli demonstrates how image dynamics can directly influence refractive error development.

Together, these articles converge on a shared insight: myopia is a complex, multi-system condition best understood through integrated perspectives. We hope this Research Topic serves as both a scientific resource and a catalyst for future interdisciplinary advances in understanding myopia. We hope this Research Topic will serve as a valuable resource for researchers, clinicians, and policymakers working toward the shared goal of reducing the global burden of myopia.

